# Doctors Who Are Using E-mail With Their Patients: a Qualitative Exploration

**DOI:** 10.2196/jmir.5.2.e9

**Published:** 2003-05-15

**Authors:** Madhavi R Patt, Thomas K Houston, Mollie W Jenckes, Daniel Z Sands, Daniel E Ford

**Affiliations:** ^1^Johns Hopkins UniversityBaltimore MDUSA; ^2^University of Alabama at BirminghamBirmingham ALUSA; ^3^Beth Israel Deaconess Medical Center and Harvard Medical SchoolBoston MAUSA

**Keywords:** Electronic mail, Internet, physician-patient relations, communication

## Abstract

**Background:**

Despite the potential for rapid, asynchronous, documentable communication, the use of e-mail for physician-patient communication has not been widely adopted.

**Objective:**

To survey physicians currently using e-mail with their patients daily to understand their experiences.

**Methods:**

In-depth phone interviews of 45 physicians currently using e-mail with patients were audio taped and transcribed verbatim. Two investigators independently qualitatively coded comments. Differences were adjudicated by group consensus.

**Results:**

Almost all of the 642 comments from these physicians who currently use e-mail with patients daily could be grouped into 1 of 4 broad domains: (1) e-mail access and content, (2) effects of e-mail on the doctor-patient relationship, (3) managing clinical issues by e-mail, and (4) integrating e-mail into office processes. The most consistent theme was that e-mail communication enhances chronic-disease management. Many physicians also reported improved continuity of care and increased flexibility in responding to nonurgent issues. Integration of e-mail into daily workflow, such as utilization of office personnel, appears to be a significant area of concern for many of the physicians. For other issues, such as content, efficiency of e-mail, and confidentiality, there were diverging experiences and opinions. Physicians appear to be selective in choosing which patients they will communicate with via e-mail, but the criteria for selection is unclear.

**Conclusions:**

These physician respondents did perceive benefits to e-mail with a select group of patients. Several areas, such as identifying clinical situations where e-mail communication is effective, incorporating e-mail into office flow, and being reimbursed for online medical care/communication, need to be addressed before this mode of communication diffuses into most practices.

## Introduction

Effective patient-provider communication is essential to comprehensive, quality health care [1,2]. Improving physician-patient communication is increasingly recognized as an important health care issue [3]. As outlined in the Institute of Medicine (IOM) report, "Crossing the Quality Chasm," 1 of the 5 key areas in the way information technology could contribute to an improved health care delivery system includes enhanced patient and clinician communication [4]. The Institute of Medicine indicated "the health care system should be responsive at all times . . . and that access to care should be provided over the Internet, by phone . . . in addition to face-to-face visits" and that "a 2-minute email communication could meet many patients' needs more responsively and at a lower cost."

E-mail is a rapidly-growing communication medium, particularly as time limitations on the part of both providers and patients increase. Technically-minded, electronically-equipped, health care consumers have accelerated the demand for e-mail access to their providers [5]. Although more than 100 million Americans now use the Internet (and many access health information), few doctors communicate with their patients through e-mail [6,7]. Despite its potential to improve both the quality and efficiency of health services delivery [8], the use of e-mail communication has not been widely adopted by many clinicians. There is still a wide gap between patients' desire for e-mail communication with their healthcare providers and providers' acceptance of this electronic patient-centered communication [6,9]. Yet other data indicate that physicians are more optimistic than patients about the potential for e-mail use, particularly as it impacts the doctor-patient relationship [10]. Concerns regarding additional time demands that e-mail communication may impose [10,11] and trouble finding time to connect to the Internet are reported by many clinicians [12]. Also, due to the asynchronous nature of electronic communication, e-mail may not be an effective medium for some patient-physician interactions [13].

There are several unanswered questions regarding this method of communication that must be answered before widespread use can be expected. How does this new technology impact the patient-physician relationship? For what problems can e-mail be used most effectively? How do physicians respond to patients' desire for electronic communication? How do they integrate this new technology into their practice? There is little research that documents how clinicians who use e-mail daily view and use e-mail communication, or documents its impact on medical practice. Some of these questions may not be fully answered until the diffusion of this technology becomes widespread. To begin to understand and generate hypotheses regarding the potential benefits and limitations of e-mail communication with patients, we explored the experiences of physicians who are currently frequent users of e-mail communication in their clinical practice. By identifying early adopters of this innovation in physician-patient communication and using methods akin to appreciative inquiry (which attempts to look at systems to find out what is currently working, and thus what is, potentially, the future ideal) we hoped to better understand how and why electronic mail is currently being used in "real-world" practices, and to perceive how the technology may be successfully used in the future.

## Methods

### Sample

We identified a sample of "Internet savvy" physicians frequently using e-mail with patients through the national convenience sample of members of Physicians' Online [14], an Internet-based professional information and communication portal limited to physicians currently practicing in the United States. Respondents to an Internet-based questionnaire designed to identify *frequent users*, defined as physicians who "receive one or more e-mails from patients in a typical day," were included [15]. Of these physicians, 204 indicated daily use of e-mail and 88 expressed interest in completing an in-depth phone interview.

### Conduct of Interviews

We sent an e-mail to these 88 physicians describing our study and asking them to participate in a 10 to15 minute in-depth phone interview. Nonresponders were sent up to 4 separate reminder e-mails and those who provided phone-contact information were contacted by phone. One researcher, who was blinded to physician responses in the earlier questionnaire on e-mail use and had little familiarity with e-mail communication in health care, conducted the interviews.

The interview was designed to explore the physicians' experiences and thoughts regarding e-mail through a series of open-ended questions. A general outline of topics and questions was designed by the researchers to provide a platform for participants to generate thoughts about e-mail communication. Example questions/cues included: (1) What made you start using e-mail with your patients?, (2) Can you give me examples of how e-mail affected quality of care provided, including examples of both increased and/or decreased quality of care?, (3) Tell me about problems you've encountered using e-mail. The interviewer could ask additional questions if comments or questions by the interviewee generated new ideas. The interviews were conducted from November 2000 through April 2001. A $50 honorarium was provided to the physicians for participating. We obtained verbal consent from each physician, and audio taped all interviews, which were then transcribed verbatim.

### Data Analysis

Two authors independently identified distinct comments from the transcripts and together with a third author, who has expertise in qualitative methodology, reviewed comments and developed domains and subdomains. Repeated or reworded comments of the same thought by the same participant were counted only once. Any disagreement on whether a particular segment represented a unique thought or concept was adjudicated. Domains and subdomains were agreed upon by consensus. Taxonomy of all comments was then sent to the remaining authors to be reviewed for relevance and consistency. All discrepancies were resolved by consensus.

## Results

Among the 88 physicians contacted, 52 responded. However, 7 of these were unable to participate in phone interviews during the time interval of the study. There was no response from 36 physicians despite 4 or more e-mail attempts and, if phone contact information, was provided, phone messages. We completed 45 interviews. The demographic characteristics of the participating physicians are provided in Table 1. There were no statistically-significant differences in age, gender, race, and subspecialty between the participants and the nonresponders, or by positive or negative attitudes on the earlier survey.

**Table 1:** Demographic characteristics of physicians interviewed (n = 45)

Characteristic	Percent
Age (years)	
<<35	9
35-55	73
Over 55	18
Gender	
Male	82
Female	18
Specialty	
Generalists (general internal medicine, family practice, general pediatrics, general psych, preventive medicine)	64
Specialists (internal medicine, pediatrics)	20
Surgery	7
Emergency room	2
Obstetrics/Gynecology	7
Number of daily email exchanges	
1-5	87
6-10	9
11-15	2
>>15	2
Would recommend doctor-patient e-mail communication to a colleague	
Yes	84
No	16

Almost all of the 642 comments could be grouped into 1 of 4 broad domains: (1) e-mail access and content, (2) effects of e-mail on the doctor-patient relationship, (3) managing clinical issues by e-mail, and (4) integrating e-mail into office processes. The full taxonomy is represented in Table 2. Twenty-six comments could not be classified into one of the domains. A prominent and consistent subdomain, use of e-mail for chronic-disease management, which was identified as a major finding, is summarized in Box 1. Details and examples of specific categories within the taxonomy are described below.

**Representative comments from the prominent and consistent subdomain of e-mail use for chronic disease management**

I had a guy who wrote to me specifically about his dose of Ritalin and informed me how he was doing. I wrote him back and told him what to do about adjusting his dose.

Usually, I use it with patients that have an established condition that we are managing together and I want to spare them the time and expense of an office visit for something I don't really need to do an office visit for. These are my sugars what should I do? My asthma is kicking up should I increase my steroids?

For diabetic patients with sliding scales, they can send me email with their sugar levels and they will change their treatment according to what I recommend.

Patients sending in their home blood pressures, glucose monitors, ordering tests, making sure their tests get ordered before their visit, sending information back and forth to get stuff done.

Patients sending in their home blood pressures, glucose monitors, ordering tests, making sure their tests get ordered before their visit, sending information back and forth to get stuff done

**Table 2:** Taxonomy of comments

I. E-MAIL ACCESS AND CONTENT
E-MAIL AS AN ALTERNATIVE OPTION FOR PATIENTS
E-MAIL IMPROVES ACCESSIBILITY
Direct access to provider
Increases access to patients and providers away from office setting
ISSUES ADDRESSED VIA E-MAIL
Sensitive issues
Emergencies
Inappropriate
General information
Limitations
II. E-MAIL AND THE DOCTOR-PATIENT RELATIONSHIP
CONCERN REGARDING CONFIDENTIALITY
IMPACT OF E-MAIL ON TRUST/RAPPORT
III. MANAGING CLINICAL ISSUES BY E-MAIL
USE OF E-MAIL FOR CHRONIC DISEASE MANAGEMENT
USE OF E-MAIL FOR PATIENT EDUCATION
USE OF E-MAIL FOR RX REQUESTS
USE OF E-MAIL TO IMPROVE CONTINUITY OF PATIENT CARE
Previsit information
Followup
IV. INTEGRATING E-MAIL INTO OFFICE PROCESSES
GETTING STARTED USING E-MAIL WITH PATIENTS
Acceptance by physicians and patients
Promotion and initiation of e-mail use with patients
Selection of patients
POLICIES ON HOW TO USE E-MAIL
General
Medicolegal
Reimbursement
E-MAIL AND VOLUME OF PATIENT CARE
Ability to address more issues
Concern regarding overuse of e-mail by patients
IMPACT OF E-MAIL ON PRACTICE EFFICIENCY
Increased convenience and flexibility for patients and physicians
Managing time demands of physicians and patients
Volume insufficient to notice significant change in practice
INCORPORATING E-MAIL INTO DAILY OFFICE WORKFLOW
Documentation
Technical problems - information technology related
Use of office personnel
Responding to e-mail in timely manner

### E-mail Access and Content

Many physicians considered e-mail as an alternative communication tool for patients and providers, primarily as a result of difficulties experienced with the phone system. These physicians believed that e-mail saves time spent on tracking patients down via the phone and allows more opportunity for patients to communicate with them during the busy workday and after hours. For example:

If they [patients] have a simple question that is not urgent then they don't have to wait on the phone, for example "Can I take my meds at bedtime with milk?"

The comments reflected a belief that e-mail increases physician's accessibility to patients, both by providing a direct route to the physician and by allowing continuous communication when patients are traveling. Some believed that direct access to the physician is a benefit to the patients. A few stated that this could become a potential burden for the physician if there is no triage system with nurses or other office personnel. Others expressed concern about e-mails not reaching them at all or in an untimely manner. For example:

Sometimes [the nurses] filter questions [received by phone] appropriately but sometimes they don't. With e-mail, when patients mail me a concern I get it.

I had a patient e-mail me with questions about whether he needed a tetanus shot [after an acute event] and I got the message [several days later]

There were mixed opinions regarding the potential scope of topics covered by e-mail. Most felt that e-mail provided a somewhat-anonymous medium through which many patients could discuss "sensitive" topics that they may not have otherwise discussed. Patient surrogates, ie, family members or caretakers, have also used e-mail to introduce issues the patient had been reluctant to discuss with the physician. However, some physicians stated that there are some inherent limitations to using e-mail for communicating complex issues and that using e-mail cannot be done as casually as talking on the phone or in person.

There was also divergence on the issues of urgencies or emergencies. A few physicians gave examples of when e-mail actually helped in these situations and thought that e-mail could and should be used for emergencies. However, the majority believed e-mail should be utilized for nonemergency matters. Almost all the physicians felt that e-mail is a great medium for exchange of general information, such as scheduling and general clinical questions. When confronted with difficult, vague, or inappropriate questions, physicians generally asked the patients to call the office. Physicians in favor of e-mail told us:

There are some patients who are unable to communicate verbally but who are able to put information on paper or who have become accustomed to chat rooms. With those people, I have been able to communicate much more effectively. I had one patient who e-mailed me that she had another issue to discuss with me but she hadn't brought it up earlier because she was too embarrassed to do so in front of the medical student.

Wives e-mail and tell me that their husbands are coming in and they are not going to say this but they are passing blood, etc.

Those with concerns reported:

The only thing that I am scared of honestly is when patients e-mail me with problems like "shortness of breath" or with 20 questions which they feel like I should be able to answer right away.

For me, e-mail or phone — I limit it. I use it only for getting some information. I don't even like using the phone for long communication, I ask the patient to come in.

There is a difference between seeing someone in the office and seeing them via e-mail with nothing but a name.

### E-mail and the Doctor-Patient Relationship

These physicians seemed to have mixed views regarding confidentiality and e-mail. Some physicians were not concerned about confidentiality as long as the patients were "comfortable" using e-mail, while others were concerned and did not include personal information in e-mails. An example of these concerns is:

The biggest snafu that I committed was with a patient's husband, who was having an affair, I breeched patient confidentiality, by sending information to one spouse who I thought was then giving it to the other spouse.

There was concern, among these physicians, of potential loss of trust and a negative impact on the doctor-patient relationship. When there is a loss of trust, it is difficult and takes a long time to regain. At the same time, physicians told us that e-mail communication has a positive effect on the doctor-patient relationship by increasing rapport and keeping lines of communication open. For example:

I look at e-mail as a fabulous way to establish a rapport with my patients.

With e-mail we are able to keep the lines of communication open.

Patients feel more of a one to one relationship

### Managing Clinical Issues by E-mail

Chronic disease management is one area of consistent agreement for our respondents. These physicians felt that e-mail is a very effective way of managing patients whom they know well. Many cited examples of using e-mail to manage conditions, such as diabetes, hypertension, psoriasis, and even congestive heart failure.

Many of these physicians also felt that there is great potential for exchange of educational information via e-mail, and, therefore, subsequent improvement in clinical management. They felt that e-mail is a useful educational tool. A representative comment is:

I have had a few patients [who] you don't have time in the office to give them specific details about their disease. I have compiled a list of articles or Internet sites, and I can e-mail those lists to them. It is not complete yet, it has to be categorized and organized more. But the patients love it.

One very-specific use for e-mail, stated by most physicians, is for prescription refills. There were comments, primarily from physicians who do not currently prescribe medications online, concerning the appropriateness of this, the medicolegal concerns, and the difficulty of doing this online. A few physicians who currently provide refills online also expressed these concerns. A separate site designed to deal specifically with medications was suggested as a helpful alternative. Physicians told us that they have realized that e-mail can be very effective for this aspect of patient care. For example:

A few patients have got hold of my e-mail address and have started to send me information about prescription renewal or questions. At first, I thought it was an intrusion but I realized what a time saver it was. So yes, I now use it for my regular practice as well; it has helped efficiency.

Most physicians' comments seem to reflect the idea that e-mail can improve the continuity of patient care if used for previsit information gathering and followup, particularly with test results and scheduling of tests previsit; this is primarily useful for patients with chronic diseases and for those with whom they had an ongoing relationship. Some stated that receiving preliminary information — such as basic past medical history, allergies to medications, and current medications and doses — prior to the visit saves time during the visit and allows more time to be spent on management. Physicians also felt that e-mail allows patients to communicate with them after leaving the office, particularly for clarification or asking questions they forgot during the visit.

### Integrating E-mail Into Office Processes

There were numerous concerns regarding the technical and day-to-day aspects of actually integrating e-mail communication into daily practice. Comments ranged from the general acceptance of e-mail by patients and providers to broad policy issues and technical implementation into the daily schedule. For the most part, physicians seemed to accept e-mail and felt that it is going to increase in the future. Most physicians felt that their patients who use e-mail love it. There was some ambivalence though, particularly regarding how and for what purposes it should be used. One comment described e-mail as a "double edged sword." There was also some concern regarding the potential substitution of e-mail for visits. For example:

A policy needs to be in place regarding expectations about response time, what can be asked, the types of things that would be appropriate or inappropriate, and how my e-mail would be handled if I were to go out of town.

Selection of patients for e-mail communication appears to be an issue with which physicians are grappling. Physicians appear to be selective in choosing patients whom they will communicate with via e-mail, but it is not clear, other than patient access to the Internet, what criteria they use. For example:

I have chosen my patients impromptu, people who I think can handle the task [of using e-mail].

There are a few patients who I do not know well, and e-mail in those instances, is logistically more difficult. I only give it to selected patients. I kind of pick the ones that I know won't abuse it.

Promoting and initiating e-mail with patients was also an area of confusion for these physicians. While some physicians were offering the use of e-mail to their patients, a number of physicians commented that patients initially approached them with the idea of using e-mail. A few had advertised the use of e-mail on their Web pages and business cards. There were reservations regarding getting inundated with e-mail, with current methods of advertisement, and these concerns deterred some physicians from putting their e-mail on the business card. On the other hand, there was a fear of disenfranchising patients if they did not offer e-mail.

With regard to general and medicolegal issues, the responses indicated that physicians do not have formal policies in place regarding how e-mail should be used with patients. Those who did have formal policies in place generally had a consent sheet or had their patients sign a waiver; a few physicians followed American Medical Association (AMA) guidelines [16]. Many had informal dialogue with their patients and a general implicit acceptance and belief that their patients understood that e-mail would be used in specific ways, such as for nonemergency use. Many indicated that a formal policy would be important and useful to having e-mail run smoothly, particularly addressing such issues as response time and appropriate content, including updates on progress and general medical questions.

Physicians felt that they should be reimbursed for e-mail exchanges, but were skeptical that this would happen in the near future, as they had difficulty getting reimbursed for phone consultations. Again, opinions varied. Some of the physicians did not seem to be too concerned regarding reimbursement, while others feared that this is a potential deterrent to widespread use of e-mail. The following comments are a few examples:

If no one is going to pay you for the time, it is not cost effective to use e-mail.

Unless reimbursement changes, e-mail consultation won't work.

For physicians time is money.

There were diverging comments on the impact of e-mail on volume of patient care. Some physicians reported that they were able to address more issues and take care of more patients since some of the preliminary, noncritical topics were handled by e-mail. Others reported concerns that e-mail can be redundant, overused by patients, time-consuming, and can potentially overburden physicians as e-mail use increases. This is an anticipated fear.

There were also conflicting opinions and experiences regarding the impact of e-mail on efficiency. Some stated that e-mail was more convenient, offered more flexibility and saved time. Others felt that e-mail could become an added burden, particularly if the physician is solely responsible for handing the e-mails. Most physicians felt that e-mail is more convenient and increases the flexibility of both physicians and patients in terms of addressing medical questions. The ability to communicate via e-mail outside office hours and on their schedule is viewed as an important benefit. Some examples included:

E-mail is so much more efficient, you end up knowing the patients so well by the time they come for followup, that you can ask more direct questions about what has been going on with their lives, why their blood pressure is consistently up, etc.

No matter what you do there is always limited time in the office. With e-mail the patients are unlimited with their time. They can ask me questions that they forgot to ask while they were in the office.

It is more work for the physicians. On the other hand, I can answer e-mails when I am at home, when I am eating, or whenever.

There did not appear to be a clear method of documentation. Some respondents felt that documentation by e-mail was much better and easier than by phone; others, who were struggling with how to do this efficiently and incorporate it into office flow, felt that documentation was worse with e-mail. For example:

There is much better documentation. I write my response, copy it, and put it in the medical records. In terms of efficiency, it is a wash because it takes me as much time to write an e-mail as to make a phone call.

Things are not documented as well as when patients use the phone. We have a formal system of phone calls, but not for e-mails.

Physicians were concerned about the technical aspects of using e-mail — particularly with servers malfunctioning and systems failing, inadvertently leading to missed e-mails. This appeared to be more of an anticipated concern than one frequently experienced.

Finally, there was a wide spectrum of opinions and experiences in reference to use of office personnel and colleagues using e-mail to communicate with patients. Most physicians had not fully broached the subject with their staff. Many who did felt that their staff was not prepared or interested in using e-mail. The few who had incorporated office personnel into e-mail communication met with success. Most physicians seemed to be responsible for accessing their own e-mail, even when out of town, although some respondents told us about colleagues accessing their e-mail.

## Discussion

We attempted to search for what is currently working in electronic patient-centered communication, and through that, to identify "what might be" in the future. We talked with 45 physicians who were frequently using e-mail with patients and found that most opinions regarding electronic patient-physician communication were positive. These physicians did see a benefit to using e-mail in specific situations with specific patients. Physicians reported better and more-consistent communication with patients who have chronic diseases and require frequent, small changes in management. Respondents noted several other benefits including continuity of communication with patients (particularly patients who travel), ability to respond to nonurgent issues on their own time, avoidance of phone tag with patients, and improved efficiency in certain scenarios. Drug-refill requests and dissemination of educational information, including links to reliable Internet sources, were also cited as examples of the effective use of e-mail with patients.

Despite the positive experiences, e-mail communication is not yet widespread in clinical care. We heard about a number of barriers that may be influencing this, such as uncertainty of involving office staff, potential of increased demand on physician time (particularly with overuse of e-mail by patients), difficulty incorporating e-mail into daily office work flow, generating timely responses, inappropriate or urgent content in the messages, confidentiality issues, and lack of reimbursement for this service. In previous research by Moyer et al, almost half of a sample of physicians at 2 university-based primary care clinics indicated concerns about being overwhelmed by patients' e-mails and felt that e-mail with patients would add to their workload if they used it in their clinical practice [10]. The only controlled trial implementing e-mail communication between physicians and patients revealed no significant reduction in volume of phone communication; thus supporting these physicians' concern [17]. Although most physicians did not express concern about confidentiality, those who did were very concerned. Current guidelines for physician-patient e-mail and medicolegal reports identify the potential risks to confidentiality and the importance of establishing policies for integration of technology into practice [18]. Despite existence of guidelines [16,18], physicians, for the most part, do not have established formal policies or guidelines that they use with patients regarding e-mail communication. Those who do have formal polices in place appear to have fewer concerns about content and overall use.

The respondents anticipated other problems with e-mail communication, such as reimbursement problems, logistic and technical problems (such as failing servers, lost e-mails), and medicolegal consequences of e-mail used for urgent issues, but had not frequently experienced them. The physicians in this study appeared quite concerned about the "nuts and bolts" of integrating the technology into the workflow of their clinical practice. Technology for secure communication between physician and patient has not been widely integrated with the medical record system or other office systems for scheduling or triage. Future development in this area may increase e-mail adoption. Expert opinions from prior literature have highlighted additional limitations of e-mail physician-patient communication [13]. Specifically, the asynchronous nature of the communication may not be amenable to complex diagnostic issues.

An important aspect of e-mail communication involves how physicians select patients for whom they will start e-mail communication. Criteria used to select patients for e-mail communication, such as a patient's "ability to handle it" (verbatim comment from one of the physicians), were not well defined or objective, and do not depend only on access to the Internet. Whether selection depends on the length of the doctor-patient relationship, the nature of the medical issue, educational achievement of the patient, or other factors is not clear and requires further research. E-mail communication may lead to greater, not less, inequality in access to care for certain patient groups.

Our study is limited by a relatively-small sample size. The sample was recruited from an online physician organization and is probably more Internet savvy than most physicians. However, these physicians represent several specialties and were from a wide geographic region. Because the use of electronic patient-centered communication is largely unstudied, we feel that the qualitative nature of this study has particular strengths. Our results included 642 comments from physicians across the United States who are frequent users of this technology and reflected a wide range of opinions. Many outcomes, including the selection of specific patients, the lack of concerns related to confidentiality, and the large number of anticipated but unrealized technical problems, were not perceived as potential major themes prior to collecting these data. We believe these qualitative methods have provided useful pilot data for future studies of the feasibility of dissemination and potential impact of this technology.

These physician respondents did perceive benefits to e-mail with a select group of patients. Through this study we identified several areas of future research. These include: developing criteria for selecting patients to use e-mail; increasing dissemination of formal guidelines regarding e-mail use; improving incorporation into office flow; use of office personnel to manage e-mail; clarifying medicolegal consequences; and mechanisms for reimbursing online medical care/communication.
